# Familial association of pancreatic cancer with other malignancies in Swedish families

**DOI:** 10.1038/sj.bjc.6605363

**Published:** 2009-10-13

**Authors:** E Hiripi, J Lorenzo Bermejo, X Li, J Sundquist, K Hemminki

**Affiliations:** 1 Division of Molecular Genetic Epidemiology, German Cancer Research Center (DKFZ) Im Neuenheimer Feld 580, 69120, Germany; 2 Karolinska Institute, Center for Family and Community Medicine, 141 83 Huddinge, Sweden; 3 Center for Primary Health Care Research, Lund University, Malmö, Sweden; 4 Stanford Prevention Research Center, Stanford University School of Medicine, California, USA

**Keywords:** familial risk, pancreatic cancer, family history

## Abstract

**Background:**

The aim of this study was to characterise the familial association of pancreatic cancer with other malignancies.

**Methods:**

Relative risks (RRs) of pancreatic cancer according to family history of cancer were calculated using the updated Swedish Family-Cancer Database, which includes over 11.5 million individuals. Estimates were based on Poisson regression. RRs of tumours for individuals with a parental history of pancreatic cancer were also estimated.

**Results:**

The risk of pancreatic cancer was elevated in individuals with a parental history of cancers of the liver (RR 1.41; 95% CI 1.10–1.81), kidney (RR 1.37; 95% CI 1.06–1.76), lung (RR 1.50; 95% CI 1.27–1.79) and larynx (RR 1.98; 95% CI 1.19–3.28). Associations were also found between parental history of pancreatic cancer and cancers of the small intestine, colon, breast, lung, testis and cervix in offspring. There was an increased risk of pancreatic cancer associated with early-onset breast cancer in siblings.

**Conclusion:**

Pancreatic cancer aggregates in families with several types of cancer. Smoking may contribute to the familial aggregation of pancreatic and lung tumours, and the familial clustering of pancreatic and breast cancer could be partially explained by inherited mutations in the *BRCA2* gene.

Pancreatic cancer is the fourth most common cause of death from cancer in Sweden (Hemminki and Li, 2003a; Linder *et al*, 2007). Prognosis for patients with this disease is very poor: the median survival time is 6 months and the 5-year survival rate is below 10% (Rulyak and Brentnall, 2004; Ghaneh *et al*, 2007; Linder *et al*, 2007). Early treatment of pancreatic cancer increases the likelihood of survival (Greenhalf and Neoptolemos, 2006). However, most patients are diagnosed at an advanced stage (Klein *et al*, 2002; Greer *et al*, 2007). Studying the familial clustering of pancreatic cancer with non-pancreatic malignancies meets two objectives: it provides information for cancer risk assessment in genetic counselling and provides clues with regard to the aetiology of the disease.

Around 4% of patients diagnosed with pancreatic cancer have parents or siblings who are also affected by pancreatic cancer (Hemminki *et al*, 2008). It has been estimated that up to 20% of the familial clustering of pancreatic cancer can be attributed to syndromes such as hereditary non-polyposis colon cancer (HNPCC), familial adenomatous polyposis and familial atypical multiple mole melanoma syndrome (Klein *et al*, 2001; Jaffee *et al*, 2002). The corresponding genes associated with these disorders are mismatch repair genes, *APC* and *CDKN2A* (Klein *et al*, 2001; Lynch and de la Chapelle, 2003). Other reported risk factors for the disease are tobacco smoking, obesity and diet rich in calories and meat (Coughlin *et al*, 2000; Chiu *et al*, 2001; Michaud *et al*, 2001; MacLeod and Chowdhury, 2006; Ghaneh *et al*, 2007).

The present population-based study used the Swedish Family-Cancer Database. An important advantage of the database is that information on familial relationships and cancers comes from registered sources of practically complete coverage, thus offering unique possibilities for precise and unbiased assessment. The risks of pancreatic cancer were estimated according to family history of the most prevalent types of cancer. Risks of non-pancreatic malignancies for individuals with a family history of pancreatic cancer were also calculated. The database has been used previously to assess the association of pancreatic cancer with parental history of cancers from 1961 to 1998 (Hemminki and Li, 2003a). The database was updated in 2006 and now includes over 1.2 million tumours diagnosed between 1958 and 2004. Compared with the previous version, the number of pancreatic cancer patients with a parental history of the disease increased from 34 to 84 patients. Using the updated database, we explored the risk of pancreatic cancer according to sibling history of cancer as well. As early age of onset has been a reported characteristic of hereditary cancer, we additionally calculated the relative risks of pancreatic cancer according to the parental/sibling age of diagnosis of cancer (Krainer *et al*, 1997; Raimondi *et al*, 2007).

## Patients and methods

Data on the incidence of pancreatic cancer between 1961 and 2004 in Sweden were derived from the Nordcan Database (Engholm *et al*, 2008). Relative risks of pancreatic and other cancers were calculated using the updated Swedish Family-Cancer Database. The population-based Swedish Family-Cancer Database was created by linking the Multigeneration Register at Statistics Sweden to the Swedish Cancer Registry (Hemminki *et al*, 2001). The Multigeneration Register includes individuals born in Sweden after 1931 and their biological parents. The Swedish Cancer Registry is based on compulsory reports about patients provided by pathologists and cytologists, who report every cancer diagnosis on surgically removed tissues, biopsies, cytological specimens, bone marrow aspirates and autopsies (Center for Epidemiology, 2004). The latest update of the database comprises more than 11.5 million individuals. Data on patients with cancer were retrieved from the Swedish Cancer Registry from 1961 to 2004. The coverage of the database is practically complete; however, some familial links are missing from offspring who were born before 1941 and who died between 1960 and 1997. The effect of the missing data is a reduced number of mortal cancers among offspring. This is unlikely to cause bias to familial studies because familial and sporadic cases would be reduced proportionately (Hemminki *et al*, 1998; Hemminki and Vaittinen, 1999). This study relies on individuals who had information available about both parents. Family history was restricted to first-degree relatives, that is, to parents and siblings.

Follow-up started from the date of birth, immigration or from 1 January 1961, whichever occurred last. Follow-up ended on the date of diagnosis of first cancer, on death, emigration or on the closing date of the study (31 December 2004), whichever came first. Cases of cancer and person-years were classified according to gender, family history of cancer, calendar year, age, socioeconomic status and geographical region. The distribution of the number of cases in each group was modelled by Poisson regression. We controlled for family history of cancer, gender, calendar year, age, socioeconomic status and geographical region in all models. In addition, dichotomous variables were created according to parental/sibling age of diagnosis with cancer (0–55; 56–71; 72 or older).

The Genmod procedure of SAS software was used for the analysis (SAS Version 9.1; SAS Institute, Cary, NC, USA). Standard Poisson regression assumes independent observations. To account for the possible overdispersion because of a clustered family structure, s.e. can be adjusted using Pearson's *χ*^2^-test, divided by degrees of freedom. The adjustment results in slightly wider confidence intervals. For example, the relative risk (RR) of pancreatic cancer for individuals with a family history of pancreatic cancer was 2.11 (95% CI=1.67–2.66) after adjustment and 2.11 (95%=CI 1.73–2.57) without adjustment. However, this procedure may be particularly sensitive to outlying observations that are expected in our large data set. Therefore, we show in this article unadjusted CIs and point to the possibility of conservative limits due to familial dependence. The tables in this study show only cancer sites with significant results or with at least 30 affected parent–offspring pairs.

Risk of pancreatic cancer in hereditary pancreatitis was analysed by identifying hospitalised cases of chronic pancreatitis through the Swedish Hospital Discharge Register, containing all hospitalisations in Sweden between 1964 and 2007, but with a nationwide coverage only since 1987 (for description, see (Hemminki *et al*, 2009)). Familial pancreatitis was defined when at least two family members were hospitalised with this disease. Pancreatic cancers in any family members were scored through the Cancer Registry. Similarly, patients hospitalised for cystic fibrosis were identified and their subsequent pancreatic cancers were retrieved from the Cancer Registry.

## Results

**Figure 1:**
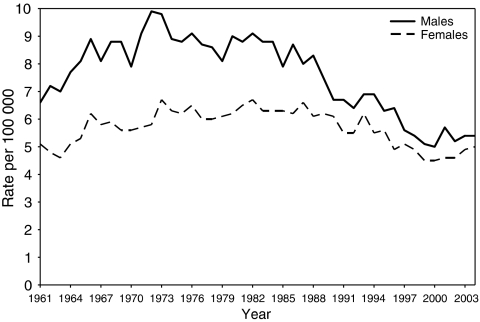
Age-standardised incidence of pancreatic cancer/100 000 individuals in Sweden in 1961–2004 (adjusted to world standard population).

Age-standardised incidence of pancreatic cancer in Sweden in the period 1961–2004 is shown in Figure 1. The incidence of pancreatic cancer in this period is stable, except from a spike in the early 1970s among men. The proportion of pancreatic cancer patients with a parent or sibling affected by the disease was 3.66% (*N*=103).

Table 1 provides the results of a multivariate Poisson regression that included a dichotomous variable for family history of pancreatic cancer diagnosed at any age, in addition to gender, calendar year, age, socioeconomic status and geographical region: women were at a slightly lower risk of pancreatic cancer compared with men (RR 0.86; 95% CI 0.75–0.97). As expected, age was found to be associated with the risk of developing pancreatic cancer. The risk of pancreatic cancer was increased among individuals with a family history of the malignancy (RR 2.11; 95% CI 1.73–2.57). Individuals with a parent diagnosed with pancreatic cancer are at an increased risk of developing pancreatic cancer (RR 1.93; 95% CI 1.55–2.40) (Table 2). There was an increased risk of pancreatic cancer among the offspring of patients with cancers of the liver (RR 1.41; 95% CI 1.10–1.81), larynx (RR 1.98; 95% CI 1.19–3.28), lungs (RR 1.50; 95% CI 1.27–1.79) and kidneys (RR 1.37; 95% CI 1.06–1.76).

In contrast, we also examined the risk of tumours in the offspring of pancreatic cancer patients (Table 3). There was an increased risk of cancer of the small intestine (RR 1.62; 95% CI 1.02–2.55) and colon (RR 1.28; 95% CI 1.10–1.48) among those with a parental history of pancreatic cancer. Parental pancreatic cancer was associated with an increased risk of melanoma in the offspring (RR 1.25; 95% CI 1.10–1.42). The offspring of patients with pancreatic cancer were at a higher risk of cancers of the liver (RR 1.52; 95% CI 1.17–1.98), lung (RR 1.18; 95% CI 1.02–1.36) and the breast (RR 1.21; 95% CI 1.13–1.31). Women with a parental history of pancreatic cancer had an increased risk of developing cervical cancer (RR 1.44; 95% CI 1.18–1.76). Sons of patients with pancreatic cancer had an elevated risk of cancers of the testis (RR 1.35; 95% CI 1.06–1.73) and other male genital organs (RR 1.88; 95% CI 1.06–3.34). The risk of Hodgkin's disease was elevated among those individuals whose parent was diagnosed with pancreatic cancer after the age of 71 years (RR 1.67; 95% CI 1.04–2.69). Parental history of pancreatic cancer after the age of 71 years increased the risk of endometrial cancer in women (RR 1.33; 95% CI 1.04–1.72).

Sibling history of pancreatic cancer tripled the risk of pancreatic cancer (RR 3.26; 95% CI 2.07–5.12) (Table 4). The risk of pancreatic cancer was elevated among siblings of patients with cancers of the oesophagus (RR 3.39; 95% CI 1.69–6.79) and liver (RR 2.07; 95% CI 1.15–3.75). Almost all siblings were diagnosed with cancer of the oesophagus or of the liver after the age of 55 years. Pancreatic cancer risk was elevated among those whose sibling had cancer of the connective tissue (RR 3.12; 95% CI 1.62–6.00). The risk of pancreatic cancer was increased among individuals with a sibling history of male genital organs (RR 3.57; 95% CI 1.15–11.1). The risk of pancreatic cancer was elevated among those whose sibling had melanoma of the eye (RR 3.26; 95% CI 1.05–10.1). An individual's pancreatic cancer risk was modified by his/her sibling's age of diagnosis of cancers at three sites. An increased risk of pancreatic cancer was noted only for those whose sibling was diagnosed before the age of 56 years with cancers of the breast (RR 1.37; 95% CI 1.06–1.77) and lung (RR 1.74; 95% CI 1.01–3.00). Similarly, there was an increased risk of pancreatic cancer among those whose brother had prostate cancer before the age of 56 years (RR 2.36; 95% CI 1.23–4.54).

To establish the possible association of familial pancreatic cancer with known syndromes that show pancreatic cancer, patients were identified from the Hospital Discharge Register. A total of 1916 patients with chronic pancreatitis were identified and 66 of them were familial cases; 25 pancreatic cancer cases were identified among all patients but none belonged to the familial pancreatitis group. A total of 774 cystic fibrosis patients were identified in the Hospital Discharge Register but none was diagnosed with pancreatic cancer.

## Discussion

Studying the familial aggregation of pancreatic cancer offers a unique opportunity to advance our understanding of pancreatic cancer development. An advantage of this study was that data on familial relationships were obtained from registered sources and tumour diagnoses were histologically confirmed, excluding any type of recall bias. The updated Swedish Family-Cancer Database permitted us to study pancreatic cancer risk not only according to parental but also according to sibling history of cancer.

The small estimated proportion of familial cases of pancreatic cancer in our dataset (3.66%) was in accordance with earlier calculations (Hemminki *et al*, 2008).

Familial aggregation of cancer can be because of a shared genetic background or because of common environmental exposures within families. Risks for concordant and discordant cancers in spouses have been estimated elsewhere to quantify cancer risks from the shared environment (Hemminki and Jiang, 2002). That study was restricted to spouses who had one or more children in common and who lived together for at least 15 years after the first child's birth. The analysis showed no increased pancreatic cancer risk among spouses of pancreatic cancer patients (Hemminki and Jiang, 2002). In agreement with previous studies, our analysis confirmed an increased risk of pancreatic cancer among individuals with a family history of pancreatic cancer (Hemminki and Li, 2003a; McWilliams *et al*, 2005). The fact that the risk of pancreatic cancer associated with sibling history was higher might indicate a recessive mode of inheritance. The greater risk in siblings than in offspring could also suggest that anticipation is operative in familial pancreatic cancer (Lerch, 2006; McFaul *et al*, 2006).

Previous studies reported an increased risk of pancreatic cancer in the offspring of patients with cancers of the pancreas, rectum and lungs (Hemminki and Li, 2003a). Associations between pancreatic cancer and cancers of the colon, breast and liver have also been described (Hemminki and Li, 2003b; Hemminki and Chen, 2004; Lorenzo Bermejo and Hemminki, 2004). Our analysis confirmed the above-mentioned findings and showed that cancers of the cervix, testis and of other male genital organs may be associated with pancreatic cancer; however, additional studies are necessary to confirm these associations.

Cancer syndromes and mutations could contribute to the observed aggregation of pancreatic cancer with other cancers. Individuals with HNPCC have an increased risk of carcinoma of the endometrium, ovary, stomach, small bowel, pancreas and brain (Lynch and de la Chapelle, 2003). Association of pancreatic cancer in our study with colon and small intestinal cancers may indicate the presence of HNPCC in some families in the Swedish Family-Cancer Database (Lynch and de la Chapelle, 2003). Inherited mutations of the *CDKN2A* tumour suppressor gene have been reported in pancreatic cancer patients with a family history of melanoma (Goldstein *et al*, 1995; Vasen *et al*, 2000). The *BRCA2* gene might also be involved in the observed aggregations of pancreatic cancer with other cancers (for example, breast, prostate). Mutations of *BRCA2* were implicated in cancers of the pancreas (Thorlacius *et al*, 1996; Couch *et al*, 2007) and breast (FitzGerald *et al*, 1996; Gayther and Ponder, 1997). Germ line mutations of BRCA2 were also linked to early-onset prostate cancer (Agalliu *et al*, 2007). Our analysis showed that early age of onset of sibling breast cancer increased an individual's risk of pancreatic cancer. We also found that there was an increased risk of pancreatic cancer associated with early-onset prostate cancer in siblings. The risk of breast cancer was also increased among offspring of pancreatic cancer patients.

An approximately twofold increased risk for pancreatic cancer was observed among ever smokers compared with non-smokers (Fuchs *et al*, 1996; Muscat *et al*, 1997; Hassan *et al*, 2007). Smoking is also a major risk factor of lung, oesophagus and kidney cancers (Chiu *et al*, 2001; Ghaneh *et al*, 2007). In accordance with previous studies, we found that pancreatic cancer was associated with other tobacco-related cancers (oesophagus, lung and kidney) (Hemminki and Li, 2003a; Shen *et al*, 2006). In addition, reverse analysis showed that early onset of pancreatic cancer in parents was positively associated with the risk of lung cancer in offspring.

Hereditary pancreatitis and cystic fibrosis are known but rare risk factors of pancreatic cancer (Howes *et al*, 2004; Maisonneuve *et al*, 2007; Rebours *et al*, 2009). However, using the nation-wide Hospital Discharge Register for case identification, no pancreatic cancer could be linked to these causes. In a European study on 418 patients from hereditary pancreatitis families, 26 (6%) were diagnosed with pancreatic cancer but close to 70% of the cancer cases were diagnosed 50 or more years after the diagnosis of pancreatitis (Howes *et al*, 2004). However, our maximal follow-up time was 43 years, of which only 21 years were with a full national coverage, decreasing the chances of finding a relationship between the two diseases. Pancreatic cancer is a rare complication after cystic fibrosis and the risk of estimation has been based on international case collections (Maisonneuve *et al*, 2007).

A potential limitation of our study was the unavailability of information on other potential risk factors of pancreatic cancer such as tobacco smoking, alcohol consumption and diet. It is important to note that some of the significant associations could be attributed to chance as well. Our investigation was also limited by the small number of tumours at some locations. The problem could be alleviated when future updates of the Swedish Family-Cancer Database are available.

The present results on the aggregation of pancreatic cancer with other cancers in Swedish families suggest that pancreatic cancer shares a genetic and/or environmental aetiology with cancer at several sites. The results of this study demonstrated a familial association of pancreatic tumours with cancers of the lung, liver and kidney. An association was also found between pancreatic cancer in parents and melanoma in offspring. Individuals with a parental history of pancreatic cancer showed an increased risk of small intestine, colon, lung, breast, testicular and cervical cancers. There was an elevated risk of pancreatic cancer among those whose sibling had lung cancer before the age of 56 years. Some of the observed associations might be related to smoking and mutations in genes such as *BRCA2*.

## Figures and Tables

**Table 1:** Relative risk of pancreatic cancer according to sex, age and family history of pancreatic cancer

**Covariate**	**Level**	***N***	**RR (95% CI)**
Gender	Females	1314	**0.86** (0.75–0.97)
	Males	1502	1.00 (ref.)
Age of diagnosis (years)	Before 50	507	**0.03** (0.03–0.04)
	50–54	502	**0.32** (0.28–0.35)
	55–59	751	**0.60** (0.55–0.66)
	60 and above	1056	1.00 (ref)
Family history	Yes	103	**2.11** (1.73–2.57)
of pancreatic cancer	No	2713	1.00 (ref.)

Bold signifies *P*<0.05.

**Table 2:** Relative risks of pancreatic cancer (RR) for the offspring of individuals affected by cancer

	**Parental age of diagnosis with cancer**							
	**Any age**		**0–55**		**56–71**		**72+**	
**Parental cancer site**	***N***	**RR (95% CI)**	***N***	**RR (95% CI)**	***N***	**RR (95% CI)**	***N***	**RR (95% CI)**
Upper aerodigestive tract	25	1.06 (0.71–1.56)	5	1.79 (0.75–4.31)	13	1.15 (0.67–1.99)	7	0.74 (0.35–1.55)
Oesophagus	11	0.98 (0.54–1.77)			5	0.98 (0.41–2.35)	5	0.93 (0.39–2.24)
Stomach	82	1.09 (0.87–1.36)	8	1.25 (0.63–2.51)	36	1.09 (0.78–1.51)	38	1.08 (0.78–1.48)
Small intestine	3	0.53 (0.17–1.64)						
Colon	109	1.03 (0.85–1.25)	13	1.62 (0.94–2.80)	42	1.02 (0.75–1.39)	54	0.97 (0.74–1.27)
Rectum	73	1.23 (0.97–1.55)	6	1.20 (0.54–2.67)	28	1.13 (0.78–1.65)	39	1.33 (0.97–1.82)
Liver	63	**1.41** (1.10–1.81)	4	1.67 (0.63–4.45)	13	0.72 (0.42–1.24)	46	**1.90** (1.42–2.55)
Pancreas	84	**1.93** (1.55–2.40)	10	**3.32** (1.78–6.18)	41	**2.19** (1.61–2.98)	33	**1.54** (1.09–2.17)
Larynx	15	**1.98** (1.19–3.28)			8	1.96 (0.98–3.92)	6	**2.41** (1.08–5.37)
Lung	136	**1.50** (1.27–1.79)	20	**2.52** (1.62–3.92)	56	1.26 (0.96–1.64)	60	**1.58** (1.22–2.04)
Breast	156	1.12 (0.95–1.32)	27	1.02 (0.70–1.49)	76	1.25 (1.00–1.58)	53	1.05 (0.80–1.37)
Cervix uteri	18	0.68 (0.43–1.08)	9	0.71 (0.37–1.37)	8	0.81 (0.41–1.63)		
Endometrium	41	1.19 (0.87–1.62)	10	1.61(0.86–2.99)	21	1.27 (0.82–1.95)	10	0.88 (0.47–1.64)
Ovary	22	0.72 (0.47–1.09)	6	0.94 (0.42–2.08)	10	0.65 (0.35–1.21)	6	0.68 (0.31–1.52)
Prostate	197	1.02 (0.88–1.18)	8	1.08 (0.54–2.17)	65	0.99 (0.77–1.26)	124	1.03 (0.86–1.23)
Testis	2	1.16 (0.29–4.64)						
Other male genital organs	3	1.30 (0.42–4.02)						
Kidney	60	**1.37** (1.06–1.76)	7	1.36 (0.65–2.85)	27	1.27 (0.87–1.86)	26	**1.51** (1.03–2.22)
Urinary organs	66	1.03 (0.81–1.32)	4	0.82 (0.31–2.18)	27	1.02 (0.70–1.49)	35	1.09 (0.78–1.52)
Melanoma	34	1.30 (0.92–1.82)	9	1.66 (0.86–3.21)	16	1.54 (0.94–2.52)	9	0.89 (0.46–1.70)
Squamous cell skin	47	0.88 (0.66–1.18)	2	0.77 (0.19–3.08)	13	0.92 (0.53–1.59)	32	0.88 (0.62–1.24)
Eye	2	0.61 (0.15–2.43)						
Nervous system	33	1.00 (0.71–1.41)	5	0.70 (0.29–1.69)	20	1.21 (0.78–1.87)	8	0.89 (0.45–1.79)
thyroid gland	15	1.53 (0.92–2.54)	4	1.77 (0.67–4.73)	8	1.82 (0.91–3.65)	3	0.98 (0.32–3.03)
Endocrine glands	28	1.41 (0.97–2.05)	6	1.87 (0.84–4.17)	12	1.27 (0.72–2.23)	10	1.44 (0.77–2.68)
Connective tissue	10	1.20 (0.64–2.23)	2	1.57 (0.39–6.27)	6	1.80 (0.81–4.02)	2	0.54 (0.14–2.17)
Non-Hodgkin's lymphoma	31	0.86 (0.60–1.23)	3	0.84 (0.27–2.61)	12	0.83 (0.47–1.46)	16	0.89 (0.55–1.46)
Hodgkin's disease	8	1.33 (0.66–2.66)			3	1.06 (0.34–3.29)	4	2.27 (0.85–6.06)
Leukaemia	34	0.88 (0.63–1.24)	4	0.98 (0.37–2.61)	11	0.67 (0.37–1.22)	19	1.06 (0.68–1.67)

Bold signifies *P*<0.05.

**Table 3:** Relative risk of tumours (RR) for the offspring of individuals affected by pancreatic cancer

	**Parental age of diagnosis with pancreatic cancer**							
	**Any**		**0–55**		**56–71**		**72+**	
**Offspring cancer site**	***N***	**RR (95% CI)**	***N***	**RR (95% CI)**	***N***	**RR (95% CI)**	***N***	**RR (95% CI)**
Upper aerodigestive tract	60	1.25 (0.97–1.61)	6	1.41 (0.63–3.15)	35	**1.63** (1.16–2.27)	19	0.87 (0.55–1.36)
Salivary glands	18	**2.06** (1.29–3.29)			9	**2.17** (1.13–4.20)	9	**2.44** (1.26–4.71)
Oesophagus	21	1.16 (0.75–1.79)	2	1.63 (0.41–6.52)	15	**1.90** (1.14–3.16)	4	0.45 (0.17–1.19)
Stomach	47	1.13 (0.85–1.51)			25	1.37 (0.93–2.03)	21	1.05 (0.69–1.63)
Small intestine	19	**1.62** (1.02–2.55)			5	0.95 (0.39–2.28)	13	**2.38** (1.38–4.13)
Colon	182	**1.28** (1.10–1.48)	15	1.34 (0.81–2.23)	79	**1.27** (1.01–1.58)	88	**1.29** (1.04–1.60)
Rectum	98	1.11 (0.91–1.36)	5	0.79 (0.33–1.89)	51	**1.33** (1.01–1.75)	42	0.98 (0.72–1.32)
Liver	58	**1.52** (1.17–1.98)	5	1.80 (0.75–4.33)	28	**1.69** (1.16–2.46)	25	1.34 (0.90–1.99)
Pancreas	84	**1.93** (1.55–2.40)	10	**3.32** (1.78–6.18)	41	**2.19** (1.61–2.98)	33	**1.54** (1.09–2.17)
Larynx	15	1.26 (0.75–2.10)			7	1.32 (0.63–2.79)	7	1.22 (0.58–2.58)
Lung	191	**1.18** (1.02–1.36)	19	**1.66** (1.06–2.61)	82	1.17 (0.94–1.45)	90	1.13 (0.92–1.39)
Breast	726	**1.21** (1.13–1.31)	75	**1.32** (1.05–1.66)	361	**1.33** (1.20–1.48)	290	1.09 (0.97–1.22)
Cervix uteri	101	**1.44** (1.18–1.76)	13	1.35 (0.78–2.33)	55	**1.61** (1.24–2.10)	33	1.28 (0.91–1.81)
Endometrium	107	1.18 (0.97–1.42)	5	0.80 (0.33–1.91)	41	1.05 (0.77–1.43)	61	**1.33** (1.04–1.72)
Ovary	104	1.20 (0.99–1.45)	11	1.30 (0.72–2.35)	51	1.30 (0.99–1.71)	42	1.09 (0.80–1.48)
Prostate	316	1.01 (0.90–1.12)	11	0.67 (0.37–1.20)	151	1.12 (0.96–1.32)	154	0.94 (0.80–1.10)
Testis	65	**1.35** (1.06–1.73)	8	1.04 (0.52–2.07)	38	**1.57** (1.14–2.16)	19	1.22 (0.78–1.92)
Other male genital organs	12	**1.88** (1.06–3.34)			5	1.70 (0.71–4.12)	7	**2.43** (1.15–5.13)
Kidney	67	0.98 (0.77–1.25)			31	1.02 (0.72–1.45)	35	1.09 (0.78–1.51)
Urinary organs	110	1.11 (0.92–1.34)	8	1.09 (0.55–2.19)	47	1.08 (0.81–1.44)	55	1.15 (0.89–1.51)
Melanoma	241	**1.25** (1.10–1.42)	32	**1.47** (1.04–2.08)	133	**1.47** (1.24–1.74)	76	0.96 (0.77–1.21)
Squamous cell skin	65	1.06 (0.83–1.35)	5	1.02 (0.43–2.46)	27	0.99 (0.68–1.45)	33	1.13 (0.81–1.60)
Eye	5	0.78 (0.32–1.88)			2	0.68 (0.17–2.71)	3	1.06 (0.34–3.29)
Nervous System	151	0.98 (0.84–1.15)	21	1.13 (0.74–1.74)	61	0.84 (0.66–1.09)	69	1.10 (0.81–1.40)
Thyroid gland	35	0.83 (0.59–1.16)	8	1.49 (0.74–2.97)	14	0.69 (0.41–1.17)	13	0.80 (0.46–1.38)
Endocrine glands	93	**1.28** (1.04–1.57)	15	**1.96** (1.18–3.26)	40	1.19 (0.87–1.62)	38	1.22 (0.89–1.68)
Connective tissue	25	0.99 (0.67–1.48)	3	0.99 (0.32–3.06)	9	0.76 (0.39–1.46)	13	1.28 (0.74–2.21)
Non-Hodgkin's lymphoma	115	1.14 (0.94–1.37)	14	1.46 (0.87–2.47)	51	1.11 (0.84–1.46)	50	1.10 (0.83–1.45)
Hodgkin's disease	34	1.16 (0.82–1.62)	6	1.35 (0.61–3.02)	11	0.77 (0.43–1.39)	17	**1.67** (1.04–2.69)
Leukaemia	88	1.03 (0.84–1.27)	10	1.03 (0.56–1.92)	45	1.13 (0.85–1.52)	33	0.90 (0.64–1.27)

Bold signifies *P*<0.05.

**Table 4:** Relative risk of pancreatic cancer (RR) diagnosed in siblings of individuals affected by cancer

	**Age of diagnosis of siblings with cancer**					
	**Any age**		**0–55**		**56–71**	
**Sibling cancer site**	***N***	**RR (95% CI)**	***N***	**RR (95% CI)**	***N***	**RR (95% CI)**
Upper aerodigestive tract	8	1.28 (0.64–2.57)	6	2.03 (0.91–4.53)	2	0.61 (0.15–2.45)
Oesophagus	8	**3.39** (1.69–6.79)	2	3.16 (0.79–12.6)	6	**3.49** (1.57–7.77)
Stomach	9	1.35 (0.70–2.59)	3	1.13 (0.36–3.50)	6	1.51 (0.68–3.37)
Colon	18	0.96 (0.60–1.53)	7	0.96 (0.46–2.01)	11	0.96 (0.53–1.74)
Rectum	13	1.09 (0.63–1.89)	3	0.69 (0.22–2.15)	10	1.32 (0.71–2.46)
Liver	11	**2.07** (1.15–3.75)			10	**2.88** (1.55–5.36)
Pancreas	19	**3.26** (2.07–5.12)	9	**5.06** (2.63–9.74)	10	**2.47** (1.33–4.59)
Larynx	2	1.11 (0.28–4.46)				
Lung	25	1.09 (0.73–1.61)	13	**1.74** (1.01–3.00)	12	0.77 (0.44–1.37)
Breast	88	1.19 (0.96–1.48)	59	**1.37** (1.06–1.77)	29	0.95 (0.66–1.38)
Cervix uteri	7	0.79 (0.38–1.65)	6	0.79 (0.35–1.75)		
Endometrium	18	1.50 (0.95–2.39)	8	1.68 (0.84–3.36)	10	1.39 (0.75–2.59)
Ovary	11	0.95 (0.52–1.71)	9	1.26 (0.66–2.43)	2	0.45 (0.11–1.82)
Prostate	47	1.19 (0.89–1.59)	9	**2.36** (1.23–4.54)	38	1.07 (0.77–1.47)
Testis	4	1.09 (0.41–2.90)	3	0.88 (0.28–2.72)		
Other male genital organs	3	**3.57** (1.15–11.1)	3	**6.48** (2.09–20.1)		
Kidney	11	1.17 (0.65–2.11)	8	1.86 (0.93–3.73)	3	0.59 (0.19–1.84)
Urinary organs	17	1.24 (0.77–2.00)	9	1.64 (0.85–3.16)	8	0.98 (0.49–1.96)
Melanoma	19	0.94 (0.60–1.48)	13	0.98 (0.57–1.69)	6	0.88 (0.39–1.95)
Squamous cell skin	10	1.31 (0.70–2.44)	4	1.31 (0.49–3.49)	6	1.31 (0.59–2.93)
Eye	3	**3.26** (1.05–10.1)	3	**4.70** (1.52–14.6)		
Nervous system	15	0.88 (0.53–1.45)	10	0.92 (0.49–1.71)	5	0.80 (0.33–1.93)
Endocrine glands	13	1.38 (0.80–2.39)	5	0.81 (0.34–1.95)	8	**2.51** (1.25–5.02)
Connective tissue	9	**3.12** (1.62–6.00)	5	**2.70** (1.12–6.50)	4	**3.87** (1.45–10.3)
Hodgkin's disease	3	1.08 (0.35–3.36)	3	1.25 (0.40–3.89)		
Non-Hodgkin's lymphoma	10	0.76 (0.41–1.41)	6	0.87 (0.39–1.95)	4	0.63 (0.24–1.69)
Leukaemia	8	0.88 (0.44–1.75)	7	1.54 (0.73–3.24)		

Bold signifies *P*<0.05.
